# Supplementary material for: Are carbon emissions trading and green financial instruments synergistic? -Comprehensive quantitative research based on content analysis
Source: PLoS One. 2024 Mar 7;19(3):e0298601. doi: 10.1371/journal.pone.0298601 (PMC10919601; doi:10.1371/journal.pone.0298601)
Supplement: S1 Data — (ZIP) [file pone.0298601.s001.zip › Data/地级市Z_F工作报告.zip!/╔┬╬≈/═¡┤¿╩╨/└┤╫╘╣1⁄2╓┌║┼í╛┬φ┐╦╩2╛▌═°í┐_2011.docx]

各位代表：

　　现在，我代表市人民政府向大会作工作报告，请各位代表连同《铜川市国民经济和社会发展第十二个五年规划纲要（草案）》一并审议，并请市政协各位委员和其他列席人员提出意见。

　　一、“十一五”经济社会发展回顾

　　过去的五年，在省委、省政府和市委的正确领导下，我们坚持以邓小平理论和“三个代表”重要思想为指导，深入贯彻落实科学发展观，努力克服国际金融危机冲击和各种自然灾害的不利影响，抓住用好资源型城市转型等历史机遇，大力实施项目带动、工业强市和可持续发展战略，全力以赴促发展，千方百计保民生，圆满完成了“十一五”各项目标任务。

　　——经济保持快速增长，综合实力显著提升。全市主要经济指标增速连续八年位居全省前列。2010年实现生产总值187.73亿元，增长15.6%，增速居全省第二位，是“十五”末的2.7倍。全社会固定资产投资完成117.23亿元，增长33%，增速居全省第三位，五年累计完成369.55亿元，是前十个五年计划总和的1.6倍，投资拉动经济增长保持在8个百分点以上。财政总收入28.28亿元，增长34.3%，增速居全省第三位，是“十五”末的4.5倍；其中地方财政收入13.75亿元，增长36.5%，增速居全省第三位，是“十五”末的4.6倍，支撑发展和改善民生的能力极大增强。在2010年度全省县域经济综合考核中，宜君县的排位较上年上升了六位，耀州区上升了一位。

　　——结构调整初见成效，优势产业不断壮大。全市规模以上工业增加值年均增长19.8%，2010年完成96亿元，是“十五”末的3.2倍。新增煤炭产能650万吨、水泥产能1150万吨、电解铝产能17万吨、电力装机120万千瓦、建筑陶瓷产能1700万平方米，煤—电—铝—水泥—陶瓷联产联营循环经济产业链基本形成。电力电源、装备制造、食品、医药、旅游等接续产业蓬勃发展，占全市经济总量的比重达到30%，初步走出了一条资源综合利用、经济质量提高的资源型城市转型新路子。

　　——特色农业快速发展，农村面貌焕然一新。粮食产量稳定在23万吨以上，2010年总产达26.6万吨，创历史新高。鲜干果经济林面积由128万亩增加到202万亩，实现了农民人均5亩果，果业已成为农民增收的主渠道。以奶牛、生猪、蛋禽为主的畜牧业呈现标准化、规模化的发展势头，奶牛存栏达到1.3万头。五年建成省级新农村示范村60个，完成了150个扶贫村的整村推进建设任务，建成移民新村（点）190个，搬迁贫困人口7000户、2.8万人，实现了农村户户通电、村村通广播电视、通水泥路或柏油路的目标。解决了39.78万农村人口安全饮水问题，农村自来水普及率达到68.2%，客车通村率达到93%，农民生产生活条件显著改善。

　　——城市建设步伐加快，基础设施明显改善。以“北市区—黄堡—董家河—耀州区—新区—坡头”为骨架的大城市格局已具雏形，城市规划区面积由55平方公里扩大到110平方公里。新区开发建设不断加快，市政基础设施和服务功能日趋完善，人气得到提升，已成为全市发展最具活力的经济板块。通过实施沉陷区滑塌区居民整体搬迁、棚户区改造、漆水河河滨景观综合治理等工程，北市区人居环境显著改善。“十一五”新建公路2000公里，新增变电容量90万千伏安。城市建成区绿化覆盖率达到42.5%，自来水普及率达到96%，气化率达到72%。城镇化率由“十五”末的53%提高到61%，高于全省14.5个百分点。成功创建为省级卫生城市和省级绿化模范城市，创建国家卫生城市、全国绿化模范城市和省级园林城市工作扎实推进。

　　——节能减排力度加大，生态环境显著变化。关闭拆除了51条落后水泥生产线，淘汰落后产能478万吨，实现了水泥大市无立窑的目标。全面完成了城市规划区燃煤锅炉改造任务，新建清洁能源锅炉105台，累计给困难群众发放取暖补贴1559万元。建成了新耀污水处理厂和宜君县污水处理工程，生活污水集中处理率达到70%，出市断面水质基本达到IV类标准。2010年城市空气质量二级以上天数达到330天，比“十五”末增加101天。万元GDP能耗比“十五”末下降20%，二氧化硫和化学需氧量分别削减2.9%和8.9%。采煤沉陷区治理、210国道沿线环境综合整治取得明显成效。五年累计植树造林120万亩，治理水土流失780平方公里，森林覆盖率由“十五”末的41%提高到44.8%，绿色已成为铜川大地的主色调。

　　——改革开放不断深化，重点领域取得新进展。妥善处置了国有企业13亿元债务，完成了35户国有企业改制任务，秦岭水泥公司、东风昌河公司等企业通过资产重组摆脱了困境。非公有制经济发展势头良好，增加值占生产总值的比重由“十五”末的42.1%提高到43.7%。在全省率先完成了集体林权制度主体改革任务。医药卫生体制改革扎实推进，基层医疗机构药品“三统一”全面实施。市县级经营性文化事业单位改制任务全面完成，财政、投融资和粮食流通体制改革不断深化。市县政府机构改革任务基本完成。对外开放水平进一步提高，五年招商引资实际到位资金200亿元，是“十五”时期的4.7倍；外贸进出口总额1.2亿美元，比“十五”增长45.4%。

　　——民生工程扎实推进，人民群众得到更多实惠。民生十大工程累计投资44亿元，其中市县财政投入11.43亿元，一批事关群众切身利益的突出问题得到较好解决。五年城镇新增就业7.37万人，城镇登记失业率控制在4.5%。失业、养老、医疗、生育和工伤五项保险实现了全市统筹。破产关闭的国有、集体企业职工养老和医疗的历史遗留问题基本解决。为80岁以上高龄老人发放了生活补贴。城乡居民最低生活保障标准、农村五保供养标准不断提高，实现了应保尽保。累计建成经济适用房、廉租房等保障性住房192万平方米，2.57万户、9万人迁入新居，城镇居民人均住房面积由“十五”末的21平方米提高到30平方米。城乡居民收入年均分别增长21.9%和18.5%，2010年城镇居民人均可支配收入达到15884元，增长15.8%，超过全省平均水平189元，扭转了长期低于全省平均水平的局面；农民人均纯收入达到4789元，增长20.7%，超过全省平均水平684元。

　　义务教育进入均衡发展和“双高普九”新阶段，宜君县在全省率先建成义务教育均衡发展合格县，王益区、印台区建成“双高普九”区。铜川职业技术学院通过国家高等职业教育评估验收。开发科技成果21项，推广转化科技成果46项。铜川体育馆、图书馆和一批乡镇综合文化站、村级示范文化室、农家书屋建成投入使用。市人民医院新区分院一期工程建成运营，提前两年完成了乡镇卫生院标准化建设，在全省率先实现了社区卫生服务机构、规范化村卫生室全覆盖，新型农村合作医疗参合率达到95.6%，走在全省前列。全市人口自然增长率4.36‰，所有区县均建成为省级计划生育优质服务先进区县。城乡社区服务体系基本形成，新建城市社区服务中心（站）71个、农村社区服务中心135个。双拥共建活动深入开展，我市被命名为省级双拥模范城。

　　——民主法制建设不断加强，社会大局和谐稳定。坚持依法行政，自觉接受人大和政协的监督，五年办理人大代表建议361件、政协提案805件，办理质量不断提高。完成了“五五”普法目标任务。信访工作不断加强，耀州区、印台区、宜君县和新区实现“三无”区县创建目标。“平安铜川”创建活动扎实推进，人民群众的安全感进一步增强。重点行业和领域安全监管力度加大，市政府连续四年被省政府评为安全生产先进单位。政府职能进一步转变，廉政建设、行政监察和审计监督不断加强，各级政府办事效率、服务水平和公信力、执行力明显提高。

　　统计、人防、质监、税务、国防、地震、供销、盐务、气象、档案、外事、残联、慈善、民族宗教等工作都取得了新的成绩。

　　各位代表，“十一五”时期，是我市经济增长快、发展质量高、城乡面貌变化大、人民群众得到实惠多的五年，我们经受住了来自各方面的严峻考验，在推进改革和发展的进程中，取得了令人瞩目的成就。这些成绩的取得，是市委统揽全局、科学决策的结果，是市人大、市政协和社会各界监督支持的结果，更是全市人民同心同德、艰苦奋斗的结果。在此，我代表市人民政府，向全市广大干部群众、驻铜部队指战员、武警官兵、公安干警，向各民主党派、各人民团体、社会各界人士表示崇高的敬意和衷心的感谢！

　　“十一五”发展成绩令人鼓舞，但我们也清醒地看到，发展中仍存在一些困难和问题：经济总量小、发展不足仍然是基本市情，继续保持良好的发展势头，推动提速发展面临许多亟待破解的难题；经济结构不尽合理，接续产业发展较慢，转变发展方式、推进资源型城市转型的任务十分艰巨；城乡居民收入水平较低，县域经济实力不强，非公有制经济发展缓慢；安全生产和维护稳定的压力较大，社会管理和服务能力仍需加强；政府职能转变还不到位，一些部门办事效率有待进一步提高，工作作风有待进一步改进。

　　这些，我们要采取更加有力的措施，努力加以解决，决不辜负全市人民的重托和期望！

　　二、“十二五”的总体思路和目标任务

　　“十二五”时期，是我市加快转变经济发展方式、推进资源型城市转型的重要时期，是推动经济社会提速发展、实现富民强市目标的攻坚时期。当今世界和平、发展、合作仍是时代潮流，经济全球化深入发展；我国发展的有利条件和长期向好的趋势没有改变，工业化、城镇化、市场化、国际化深入推进；随着关天经济区和陕北能源化工基地建设步伐加快，我省将阔步迈向中等发达省份，为我们创造了良好的外部环境。中省对资源型可持续发展试点城市的支持力度不断加大，西铜同城化加速推进。科技创新孕育新的突破，产业转移蕴藏新的机遇，我市发展正处于大有作为的重要战略机遇期。

　　我们要以邓小平理论和“三个代表”重要思想为指导，深入贯彻落实科学发展观，以提速发展、富民强市为主题，以加快转变经济发展方式、推进资源型城市转型为主线，以保障和改善民生为出发点和落脚点，继续实施项目带动、工业强市和可持续发展战略，着力破解城乡居民收入水平较低、接续产业发展动力不足、非公有制经济发展缓慢、第三产业发展滞后四大难题，提升壮大优势产业，做大做强接续产业，大力发展第三产业，继续深化改革开放，推进城乡统筹发展，促进社会和谐稳定，努力实现经济综合实力、人民生活水平、生态环境建设上台阶，为实现全面小康奠定坚实基础。

　　“十二五”的基本目标是：确保实现“三个翻番”（生产总值、财政收入、城乡居民收入翻一番）；力争实现“一个赶超”（城乡居民收入赶超全国平均水平）、“三个突破”（资源型城市转型、西铜同城化发展和工业园区建设取得突破）；推动“五个提升”（城镇化水平、社会管理和服务能力、社会保障水平、社会和谐程度、人民群众幸福指数有明显提升）。生产总值年均增长15%，财政收入年均增长16%，固定资产投资年均增长30%，社会消费品零售总额年均增长16%。

　　“十二五”的主要任务是：

　　坚持转型发展，推进产业优化升级。依托资源，延伸产业链，提高附加值，着力打造能源、现代建材、果品及食品加工、养生保健四大产业基地。加大后续资源勘探开发力度，切实把资源优势转化为产业优势。加快产业结构调整，培育壮大电力电源、装备制造、生物医药、光伏照明等接续产业，大力发展旅游、物流、金融、保险、会展等现代服务业。“十二五”末，接续产业增加值占工业增加值的比重达到45%，服务业增加值所占比重明显提高。

　　坚持内涵发展，加快工业园区建设。高起点、高标准做好八大工业园区规划和建设，到2015年，工业园区基础设施基本完善，产业发展初具规模，工业增加值增速高于全市6－8个百分点，园区城市化水平达到80%以上，以工业园区为承载，形成新的经济板块和城市组团。坚持西铜同城化发展战略，依托区位、资源优势和产业基础，加快西铜城际交通建设，形成交通便利化、空间一体化的发展格局；主动承接西安国际化大都市的辐射，发展面向西安的上下游关联产品和配套产业，实现资源共享、产业互补、错位发展。完善城市服务功能，优化人居环境，吸引周边地区人流、物流、资金流向铜川聚集。

　　坚持统筹发展，加速城乡一体化进程。按照“做美城市、做强县城、做大集镇、做好社区”的思路，加快城乡规划、产业发展、基础设施、公共服务、社会保障、生态建设和社会管理统筹发展。实现国家卫生城市、全国绿化模范城市、省级园林城市和省级环境保护模范城市创建目标。推进农业产业化和县域工业化、城镇化，提高县域经济实力。加快重点镇建设，使其尽快成为县域经济次中心。创新服务方式，完善服务功能，构建和谐社区。“十二五”末，全市城镇化率达到70%，建成16个区域中心镇、57个1500人以上的中心村，初步形成以城市和县城为中心、以工业园区和重点镇为支撑、以中心村为基础的城乡一体化发展框架。

　　坚持绿色发展，推进资源节约和环境保护。牢固树立“绿色、低碳、循环”的发展理念，延伸煤—电—铝—水泥—陶瓷联产联营循环经济产业链，发展生态农业、生态交通、生态旅游和生态物流业，实现可持续发展。落实水资源管理制度，提高水资源利用效益。执行最严格的耕地保护政策，耕地保有量稳定在140万亩以上。继续加大水泥粉尘、煤烟污染和水土流失治理力度。实施重点林业生态工程建设，广泛开展造林绿化活动。“十二五”末，森林覆盖率达到48%以上，万元GDP能耗和二氧化碳排放分别下降15%，主要污染物排放总量减少10%，生活污水集中处理率和生活垃圾无害化处理率达到85%以上，城市空气质量二级以上天数稳定在300天以上。

　　坚持创新发展，不断深化改革开放。营造各种市场经济主体依法平等使用生产要素、公平参与市场竞争、同等受到法律保护的体制环境，“十二五”末，非公有制经济比重达到50%。推进行政管理、社会管理体制创新，加快财税、投融资体制改革，着力消除制约发展的体制机制弊端。建立以企业为主的技术创新体系，加大科技成果开发力度，提高推广转化率。坚持“引进来”与“走出去”相结合，提高招商引资水平，拓展对外合作领域，提升经济外向度。

　　坚持和谐发展，努力改善人民生活。加大财政对民生事业的投入，实施居民收入倍增计划，城乡居民收入增速分别保持在16%和17%。扩大社会保障覆盖面，不断提高保障标准。加大保障性住房建设和棚户区、农村危房改造力度，解决低收入和特殊困难家庭的住房问题。优先发展教育事业，加快义务教育学校标准化建设，普及高中教育和学前一年免费教育，促进教育公平、均衡发展。加强老年人事业和关心下一代工作。强化基层医疗卫生服务体系建设，实现基本公共卫生服务城乡一体化目标。实施文化体育惠民工程，满足人民群众的精神文化需求。高度重视安全生产，维护社会和谐稳定。

　　以上目标任务，体现了省委、省政府对铜川发展的战略要求，表达了铜川人民对美好生活的共同期待，展示了铜川科学发展的光明前景。我们坚信，经过五年不懈奋斗，铜川的综合经济实力一定会有更大的提升，生态环境一定会发生更大的变化，人民生活一定会得到更大的改善！

　　三、全力以赴做好2011年的各项工作

　　今年是“十二五”开局之年，做好今年的工作对完成“十二五”各项目标任务至关重要。按照市委十届八次全会的部署和要求，2011年全市经济社会发展的主要预期目标是：生产总值增长15%，财政总收入、地方财政收入均增长20%，城镇居民人均可支配收入增长16%，农民人均纯收入增长18%，居民消费价格总水平涨幅控制在4%左右，人口自然增长率控制在5.5‰以内，万元GDP能耗下降3.5%，主要污染物排放指标控制在省上规定范围内。

　　（一）强力推进项目建设，确保固定资产投资快速增长

　　突出抓好重点项目建设。今年全市安排重点项目80个，总投资678.8亿元，年内完成投资91.3亿元。加快铜黄高速第二通道、陕汽零部件加工基地等37个续建项目建设进度，力争陕西体育专修学院、市体育会展中心、秦岭纯低温余热发电等33个项目早日开工建设，抓紧做好华能铜川电厂二期工程、玉皇阁湿地公园等10个项目前期工作。确保市污水处理厂升级改造、华原100万吨和矿山120万吨水泥粉磨站等23个项目建成投运，力争全社会固定资产投资完成160亿元，增长45.3%。

　　加大项目推进力度。坚持“周协调、月考核、季兑现”制度，强化项目建设的领导责任。深入开展“千名干部下企业包项目”活动，及时研究解决项目建设中遇到的困难和问题，千方百计加快项目建设进度。深入开展投资环境整治活动，严厉打击破坏投资环境的违法犯罪行为，优化项目建设环境。

　　做好项目策划和争取工作。紧盯中省产业政策和投资重点，高起点谋划一批事关全局、带动性强的重大项目，使在库项目数量保持在500个以上。认真贯彻国务院、省政府关于促进资源型城市可持续发展的若干意见，落实好与省上有关厅局签订的战略合作协议，促进一批项目挤进中省计划盘子。全年争取中省政策性资金8亿元以上。积极开展城乡建设用地增减挂钩试点，依法处置闲置土地，做好土地复垦整理工作，储备土地不低于2000亩。

　　（二）加快新型工业化进程，推动工业提速增效

　　提升壮大支柱产业。继续加强煤炭能力建设和资源就地转化，加快照金、西川、柴家沟、白石崖、秀房沟五大矿井技术改造，开工建设冶坪煤矿，做好耀西、丰邑煤田开发前期工作，力争铜铝公司4×30万千瓦煤矸石发电、国电热电联产项目开工建设，带动煤炭就地转化率提高15%以上。围绕现代建材业发展，突出抓好铝产业链延伸，建成大秦铝合金项目，力争30万吨碳素、30万吨电解铝、20万吨铝深加工项目年内开工建设。确保凤凰日产4500吨水泥熟料生产线、声威日产2500吨特种水泥生产线等项目建成投产，力争秦岭第二条、声威第三条日产4500吨水泥熟料生产线项目建成投产，新川沟日产4500吨水泥熟料生产线开工建设。积极承接东部陶瓷产业转移，努力壮大工艺陶瓷、日用陶瓷、建筑陶瓷产业规模。

　　做大做强接续产业。加快发展食品加工、电力电源、装备制造、光伏照明等产业，尽快形成新的生产能力。确保陕煤建司管件厂迁建增能、正宝矿山设备制造等项目建成投产，旺旺三期休闲食品、陕煤化PVC管材等项目达到计划进度，LED照明产业园项目开工建设。

　　加快工业园区建设。编制市级工业园区控制性规划，明确园区产业定位。加快核心区水电路气等基础设施建设，初步形成比较完备的基础设施体系。赋予园区管委会更大的管理权限。完善市级工业园区考核办法，实行园区与所在区县双向考核。今年各市级工业园区动态储备土地不低于500亩，固定资产投资不低于10亿元，力争3―5个有规模的产业性项目落地建设。

　　加强工业运行调度。切实做好煤电油气运的协调保障，及时解决企业生产经营中面临的流动资金不足等问题，促使骨干企业开足马力生产。强化企业内部管理，搞好市场营销，提高工业运行质量。全市规模以上工业增加值增长18.5%。

　　（三）大力发展现代农业，加快城镇化进程

　　发展果牧特色产业。实施粮食单产提升工程，粮食总产稳定在20万吨以上。继续抓好鲜干果基地建设和果业提质增效，全年新栽水果8万亩、干杂果3万亩，更新改造果园7万亩，新建万亩现代果业示范园区2个、千亩现代果业示范园区20个、干杂果示范园2万亩，苹果优果率达到82%以上。抓住被列入全省奶牛养殖示范市的有利机遇，扩大奶牛、肉牛、生猪的规模化、标准化养殖，创建6个省部级标准化规模养殖示范场。加大中药材基地建设力度，新发展中药材2万亩，保有量达到7万亩。实施菜篮子工程，完成5000亩设施蔬菜建设任务。

　　提高农业组织化程度。加快周陵现代农业科技园区建设步伐，加大对龙头企业的扶持力度。加快培育行业协会，壮大专业合作社和农村经纪人队伍，扶持果业专业合作社建设冷库、畜牧专业合作社建立防疫体系、蔬菜专业合作社建设净菜加工厂，切实发挥龙头企业和专业合作社的辐射带动作用。

　　狠抓水利基础设施建设。认真贯彻《中共中央国务院关于加快水利改革发展的决定》，把水利建设作为基础设施建设的优先领域，积极争取大中型水利建设项目，加快重点水源工程建设，大力推进安全水利、民生水利、生态水利。完成龙潭水库年度建设任务，实施五里镇河综合治理工程，完成七一水库和涧沟水库除险加固。抓好农田水利等农业基础设施建设，全市发展节水灌溉面积2.5万亩，治理水土流失面积150平方公里，解决农村3.5万人的安全饮水问题。

　　加强重点镇建设。抓好耀州区城乡统筹发展试点工作，搞好黄堡镇省级重点镇标准化建设。建立重点镇建设投融资平台，力争5个重点镇完成投资3亿元以上。稳妥推进有条件农村居民进城落户工作，确保完成1.79万农村居民进城落户任务。统筹做好农村水电路气和教育、卫生、文化等基础设施建设。做好扶贫开发，移民搬迁4400人，使1.5万人实现脱贫。新建县乡公路60公里、通村通组产业路60公里，不断改善农村生产生活条件。

　　（四）加快发展旅游产业，提升壮大现代服务业

　　突出抓好“一园两景区”建设。认真落实市委、市政府《关于进一步加快旅游产业发展的决定》，依托历史文化和绿色生态资源，加快大唐养生园和照金香山、药王山大景区建设。做好照金香山国家级丹霞地质公园申报工作,开工建设陕西省照金干部培训中心，积极开展建党90周年照金红色游主题活动。编制药王山大景区规划，建成药王大道，建设高标准的祭拜广场，改造提升景区基础设施，启动景区居民及有关企业搬迁工作，完成景区周边环境综合整治任务。抓好玉华宫和陈炉景区道路、绿化、亮化等配套设施建设。做好旅游宣传促销，每个区县举办有特色的旅游节庆活动，实现接待游客数量和旅游综合收入快速增长。

　　大力发展养生保健产业。按照建设养生保健休闲城市的方向，把大唐养生园作为体验养生文化的核心区，开工建设一批以养生保健为主的产业项目，吸引一批科技含量高、劳动力密集、无污染、产业附加值高的大项目落户。加强与陕西荣民集团的合作，加快大唐养生园项目建设。挖掘药王文化内涵，加快中国中药材铜川种植基地、流通基地、研发基地和检测基地建设，举办首届中国药王养生保健节，办好中国药王养生保健论坛，以节促商，把“药王”品牌所蕴含的丰富的医药、旅游、文化资源转化成经济优势。

　　加快发展商贸物流业。推进西北现代苹果物流中心、坡头工业园区建材物流园、董家河工业园区物流服务区等项目建设。加快新区商业区项目建设，完成布点规划，制定优惠政策，引进一批有实力的集团公司，开工建设1－2个四星级以上宾馆和大型购物中心，改善新区商务环境。全社会消费品零售总额达到56亿元，服务业增加值增长15.5%以上。

　　（五）加强建设和管理，打造宜居宜业城市

　　推进西铜同城化发展。加强与西安大企业、大集团和高等院校、科研机构的合作，共建产业基地和科技成果转化基地，主动承接西安科技、资金、信息、市场、人才的辐射。确保西铜高速第二通道如期建成通车，启动西铜城际铁路项目前期工作。

　　完善城市服务功能。加大北市区改造提升、南市区开发建设力度，抓好城市道路、给排水、垃圾处理、供气等市政基础设施建设。实施王家河南路改造，完成人民公园改造提升任务。开工建设新区北环路、朝阳西路和石仁坡道路改造工程，加快西环路中段、东环路中段、华夏南道及新区牡丹园、朝阳公园建设步伐，实现华原西道与西铜高速第二通道的顺畅连接。开工建设新耀连接带休闲公园和铜川植物园。启动城市集中供热工程。力争新发展天然气用户1万户。抓好110千伏柳湾智能变和35千伏棋盘变等输变电工程的建设，完成电网建设及改造投资1.5亿元，为重大工程和群众生产生活提供可靠的电力保障。

　　着力提升城市品位。加大资金投入，强化督查考核，完成创建国家卫生城市、全国绿化模范城市和省级园林城市年度任务。开展群众性爱国卫生运动，实施城市重点区域拆除、食品市场整顿、城乡旱厕改造、废品收购站（点）治理、医源性污水处理五项工程。拆除市区沿街有碍观瞻破旧建筑3万平方米，完成农村户厕改造4.5万户，新建5个综合市场，建成停车位1500个，开工建设应急避难场所3万平方米。完成210国道铜川沿线环境综合整治任务。

　　（六）抓好生态环境建设，提升可持续发展水平

　　加大节能工作力度。以节能、节材、节水、节地为重点，开展全社会资源节约活动。加强铝冶炼、水泥、煤炭、电力等高耗能行业的监管工作，确保完成节能任务。推动建筑、交通和公共机构节能工作，建筑节能改造3万平方米，城镇新建建筑节能设计、施工执行率达到100%。加快华能铜川电厂中水利用工程等项目建设，促进水资源的循环利用，万元工业增加值用水量降低0.5%。坚持最严格的耕地保护制度，节约集约利用各类土地，做到增减挂钩、占补平衡，耕地保有量143.66万亩。

　　加强环境污染防治。启动省级环境保护模范城市创建工作。继续抓好水泥粉尘污染治理，进一步加大药王山周边水泥污染整治力度，推进水泥企业清洁生产和厂区绿化美化工程，使区域环境面貌尽快得到改观。加大城中村、城乡结合部煤烟治理力度，城市空气质量二级以上天数确保达到300天、力争达到340天以上。开工建设北市区生活垃圾处理厂，确保新耀污水处理厂正常运转，市区和宜君县城垃圾无害化处理率达到87%、污水处理率达到80%。

　　加快生态工程建设。强力推进林业重点工程建设和重点区域绿化，突出抓好新耀连接带、新区环线道路和高速公路、国道、省道及铁路沿线绿化美化工作，继续实施天然林保护、三北防护林、退耕还林工程和漆水河等三条流域绿化工作，完成植树造林18万亩。

　　生态文明建设功在当代、利在千秋。我们要牢固树立节约优先、环保优先的发展理念，全力保护好铜川这一方山水，让铜川的天更蓝、山更绿、水更清！

　　（七）加强财政金融工作，强化支撑发展的能力

　　狠抓财政增收节支。加强收入征管，完善财税库横向联网系统，堵塞征管漏洞。实施非税收入收缴制度改革，除教育收费外，其它预算外收入全部纳入预算管理。抓好资源税、煤炭价格调节基金、两权价款征收工作，确保应收尽收。严格财政资金管理，完善政府一般预算、基金预算、国有资本经营预算制度，从严控制楼堂馆所建设、公务接待费用、公费出国出境和公务车辆购置费用。

　　提高财政资金使用效益。集中财力办大事，把有限的资金用在刀刃上。管好用好资源型城市转型资金和各类转移性支付资金，做到合理调度、高效使用。创新财政资金投入方式，建立健全以资本金注入、贴息、担保为主的投入机制，引导社会资本参与项目建设和优势产业发展。建立财政资金风险评估和绩效评价机制，确保财政资金安全。

　　增强金融支持能力。完善金融服务体系，吸引商业银行等金融机构来铜设立分支机构。加快融资平台建设，扩大果园权证抵押贷款试点范围，加快区县小额贷款公司组建工作，推进村镇银行和农村资金互助社试点工作。积极发展金融中介机构，支持各类担保公司发展，活跃金融市场。加强社会信用体系建设，优化金融生态环境，制定金融支持项目建设的具体措施，解决中小企业融资难问题。

　　（八）深入推进改革开放，增强经济发展活力

　　加快重点领域改革。加大国有企业改革力度，坚决清理承包租赁等企业改革中的短期行为，加快西北耐火材料厂、铜川变压器有限公司资产重组和市燃料公司改制工作，确保迈出实质性步伐。盘活黄堡工业园区企业存量资产，用好闲置土地等资源，在项目引进上实现新突破。放手发展非公有制经济，重点支持民间资本进入基础设施、资源开发、公用事业等领域，非公有制经济增加值占生产总值的比重达到45%。不断深化医药卫生体制改革，做好宜君县公立医院改革试点工作。完善集体林权配套改革，实现林业规模化、产业化经营。完成撤乡并镇工作任务。

　　加大招商引资力度。进一步完善招商引资优惠政策，围绕大企业配套、接续产业和现代服务业发展，开展有针对性的投资促进活动。加强与友好城市、中省大型企业集团、商会的联系与协作，共建产业园区和产业转移示范区。实施“走出去”战略，积极参加西洽会、厦洽会、农高会等招商活动，全年招商引资合同利用资金80亿元，到位资金40亿元，其中引进外资2000万美元以上。

　　转变外贸增长方式。加大地产品培育和扶持力度，扩大苹果、服装、电源、铝锭、葡萄酒等地产品出口，打造以宜君县为中心的植物提取物、以耀州区和印台区为中心的鲜苹果出口基地，培育禾博医药、金圣果业2户百万美元地产品出口企业，实现外贸进出口总额900万美元。

　　（九）切实保障和改善民生，维护社会和谐稳定

　　推进社会保障体系建设。认真落实企业最低工资标准和困难群众补助标准等各项政策，建立健全职工工资正常增长机制和支付保障机制。全市工伤保险参保人数达到8.3万人，生育保险参保人数达到3.6万人，城镇基本医疗保险参保率达到92%，城镇企业职工基本养老保险扩面人数达到3613人。加快新型农村养老保险和城镇居民养老保险试点工作，两年内实现城乡基本养老保险全覆盖。启动老年大学和老干部活动中心建设。加强城乡低保规范化管理，切实做到“应保尽保、应退尽退”。实行为孤儿发放基本生活费、无生活来源残疾人发放生活补贴制度。建成市流浪未成年人救助中心项目。加大城乡医疗救助力度，确保重点优抚对象全部享受医疗补助。落实国家生源地助学贷款政策，资助新录取家庭困难大学生400名、中等职业学校学生3000名、高中生1000名。

　　加大保障性住房建设力度。加快新区4个城中村和耀州区旧城改造步伐。全年新开工住房220万平方米，其中保障性住房180万平方米，商品房40万平方米，建成100万平方米，安置住房困难群众8000户，城镇人均住房面积达到32平方米。农村危房改造3200户。

　　建立促进就业的长效机制。认真落实促进就业的各项政策，扎实做好高校毕业生、农村转移劳动力、城镇就业困难人员、退伍军人就业工作。继续实施“人人技能工程”，全年劳动力转移培训和农民实用技术培训4万人次，发放小额担保贷款1.2亿元。城镇新增就业1.6万人，下岗失业人员再就业5000人，转移农村劳动力8万人，城镇登记失业率控制在4.5%以内。

　　坚持教育优先发展。投资2亿元，完成16所中小学安全工程、30所中小学的生活设施改造等年度任务。力争王益区教育创强工作通过省上评估验收。启动实施学前一年免费教育和乡镇中心幼儿园、村级幼儿园建设达标活动，新建1所城市公办幼儿园，改造提升8所乡镇中心幼儿园，学前教育毛入学率达到65%。大力发展职业教育，开工建设铜川职业技术学院二期工程，建成一个省级骨干示范专业，完成职教招生任务。全年开发科技成果3项，推广转化科技成果8项，全市科技研发投入达到1000万元。

　　提高医疗卫生服务能力。加快市人民医院新区分院二期工程建设。新型农村合作医疗参合率达到95%，补助标准由每人每年150元提高到230元，在全市所有规范化村卫生室开展新农合门诊统筹。做好地方病、重大传染病和慢性非传染性疾病防治工作，继续对65岁以上老人进行免费健康检查，在农村产妇免费住院分娩的基础上，启动实施产前免费检查。进一步落实各项计划生育政策，全面推进“母亲健康、优生促进、计生家庭创业”三大工程，全市人口自然增长率控制在5.5‰以内。

　　搞好基层文化体育建设。新建乡镇综合文化站5个、农家书屋150家，完成190个村应急广播室建设任务。实施20个行政村农民体育健身工程和7个社区体育器材配送工程，办好市第四届老年人运动会、第五届农民运动会和2011年铜川中美国际男篮对抗赛，不断满足群众多方面的精神文化需求。

　　加强和创新社会管理。强化政府社会管理职能，广泛动员和组织群众依法参与社会管理，实现政府行政管理与基层群众自治有效衔接和良性互动。全面实施“六五”普法规划。加快社会治安防控体系建设，依法打击各类违法犯罪活动，不断提高人民群众的安全感。加强反恐应急机制和装备建设，提升反恐防恐的能力和水平。重视做好国防教育，关心支持民兵预备役基础设施建设，广泛开展双拥共建活动。做好第八次村委会换届选举工作。高度重视安全生产，夯实政府的监管责任和企业的主体责任，突出抓好煤矿、道路交通、非煤矿山等重点行业和领域的安全监管，严防重特大事故发生。加强食品药品安全监管，让人民群众吃的放心、用的安心。健全重大自然灾害、突发公共安全事件应急处理机制，有效预防和处置各类突发公共事件。切实做好信访工作，深入开展“三无”区县、部门创建和“百日矛盾纠纷排查化解”活动，确保实现“一控制、两下降”目标。

　　民生连着民心，民生凝聚人心。我们一定要把保障和改善民生摆在更加突出的位置，今年新增财力的80%主要用于改善民生，切实解决好人民群众最关心最直接最现实的利益问题，让人民群众更多地分享改革发展成果！

　　四、切实加强政府自身建设

　　全面完成今年和“十二五”时期的任务，对政府工作提出了新的更高要求。我们一定要以“为民服务零距离”活动为统揽，以“抓落实促发展”专项督查年活动为契机，大力推进政府管理创新和服务创新，以政府工作提速推动经济社会发展全面提速。

　　增强创新发展能力。扎实推进学习型政府建设，加大公务员培训力度，增强各级干部敢于担当的责任意识、合作共赢的发展意识和服务发展的大局意识，力求思想适应发展变化、知识适应时代要求、能力适应工作需要，提高驾驭复杂局面和解决实际问题的能力。要积极学习天津等发达地区成功经验，立足铜川实际，创造性地借鉴和实践，不断提高谋发展、解难题、闯新路的认知水平，用开阔的视野、改革的思路、管用的机制、创新的办法，助推铜川提速发展。

　　加快转变政府职能。结合市县政府机构改革，切实转变政府管理经济的方式，发挥市场配置资源的基础性作用，加强经济调节、市场监管、社会管理和公共服务职能。继续深化行政审批制度改革，落实岗位责任制，简化审批程序，提高行政效率。加强审计监督、行政监察，健全衡量科学发展的考评机制，形成工作有抓手、绩效有评估、过失必追究的运行体系。

　　大力推进依法行政。各级政府要严格依照法定权限和程序行使权力、履行职责。坚持科学民主决策，健全重大事项公众参与、专家咨询论证和政府集体决策相结合的决策机制。完善政府新闻发布制度，推进政府信息公开，对涉及群众利益的重大决策都要进行听证，对重大民生事项实行承诺制，进一步提高政府的公信力和执行力。加强电子政务建设，推行网络政务。自觉接受人大及其常委会的依法监督、政协的民主监督和社会舆论监督，确保依法、公正、高效行使权力。

　　狠抓工作任务落实。大力弘扬求真务实精神，突出精细管理，强化目标责任考核，做到发展项目化、任务指标化、重点工作模块化。对事关全局的21项重点工作，实行模块化管理，建立强有力的工作班子，细化目标任务，明确工作时限，加大协调力度，调动各方力量，举全市之力加以推进，以重点工作的突破推动铜川提速发展。扎实开展“抓落实促发展”专项督查年活动，加大对重大决策部署、重大项目建设、统筹城乡发展、民生工程、城乡居民增收、作风建设及群众反映强烈的热点难点问题的督促检查，跟踪督办不落实的事、追究不落实的人，以强有力的督查推动各项工作任务的落实。

　　不断加强廉政建设。始终牢记“两个务必”，恪守廉洁从政若干规定。加强政府层级监督，完善行政问责制度，规范行政许可行为。实施源头治理，加强土地管理、工程建设、产权转让、政府采购等领域专项治理，切实落实收支两条线规定，以制度规范和阳光运作，堵住公共资源交易、公共资金管理等腐败易发多发领域的漏洞。坚持勤俭节约，反对铺张浪费，改进文风会风，严格控制各类检查评比和表彰，降低行政成本，建设节约型机关。

　　各位代表，铜川的发展已站在了新的历史起点上。让我们高举中国特色社会主义伟大旗帜，以邓小平理论和“三个代表”重要思想为指导，深入贯彻落实科学发展观，在中共铜川市委的坚强领导下，倍加珍惜来之不易的发展势头，倍加珍惜风正劲足的大好局面，乘势而上，奋力拼搏，全面完成2011年各项目标任务，推动经济社会提速发展、百姓更加富裕，以优异的成绩迎接中国共产党成立90周年！
